# Canopy-Mediated Shifts in Grassland Diversity and Heterogeneity: A Power Law Approach from China’s Loess Plateau

**DOI:** 10.3390/plants14193008

**Published:** 2025-09-28

**Authors:** Lili Qian, Cong Wu, Sipu Jing, Li Meng, Shuo Liu, Xiangyang Hou, Wenjie Lu, Xiang Zhao

**Affiliations:** 1 College of Grassland Science, Shanxi Agricultural University, Taigu, Jinzhong 030801, China; qianlili2012@163.com (L.Q.); jing_qingqiu@163.com (C.W.); 15619777962@126.com (S.J.); 18219916330@163.com (L.M.); 15896087586@163.com (S.L.); houxy16@vip.126.com (X.H.); 2 Shanxi Key Laboratory of Grassland Ecological Protection and Native Grass Germplasm Innovation, Taigu, Jinzhong 030801, China; 3 Key Laboratory of Model Innovation in Forage Production Efficiency, Ministry of Agriculture and Rural Affairs, Taigu, Jinzhong 030801, China

**Keywords:** species–area relationship, power law model, agro-pastoral ecotone, biodiversity, spatial pattern, ecosystem

## Abstract

This study investigates the spatial heterogeneity and species diversity of grassland vegetation in the agro-pastoral ecotone of China’s Loess Plateau, integrating Taylor’s power law model with the minimum area concept to address scale-dependent ecological patterns. Field surveys were conducted across four vegetation types: small-leaf poplar forest (SP), pine–caragana mixed forest (PC), caragana shrubland (RC), and saline grassland (SG). Nested quadrats (0.25–8 m^2^) were used to establish species–area relationships (SARs), while binary occurrence frequency data fitted to Taylor’s power law quantified spatial heterogeneity parameters (*δ_i_*, *δ_c_*, *CACD*) and derived diversity indices (*H*′, *J*′, *D*). the results showed that species composition differed significantly among vegetation types, with RC exhibiting the highest richness (25 species) and SG the lowest (12 species). SAR analysis showed distinct *z*-values: SP had the lowest *z* (0.14), indicating minimal area effects and high homogeneity, while SG had the highest area sensitivity. Spatial heterogeneity (*δ_c_*) was highest in RC and lowest in SP. Over 82.5% of herb-layer species exhibited aggregated distributions (*δ_i_* > 0). The dominant species *Leymus secalinus* (Georgi) Tzvelev shifted from regular (*δ_i_* < 0) under SP/SG to aggregated (*δ_i_* > 0) under PC/RC. Diversity metrics peaked in PC plots (highest *H*′ and richness, lowest dominance), whereas SP showed high dominance but low diversity. *CACD* values (critical aggregation diversity) were maximized under SG. The integration of power law modeling and minimum area analysis effectively captures scale-dependent vegetation patterns. Pine–caragana mixed forests (PC) optimize biodiversity and spatial heterogeneity, suggesting moderated canopy structures enhance ecological stability. These findings provide a theoretical basis for sustainable grassland management in ecologically sensitive agro-pastoral zones.

## 1. Introduction

Vegetation serves as the core carrier of structure and function within terrestrial ecosystems, with its community composition and spatial patterns profoundly regulating biodiversity maintenance, productivity formation, and ecosystem stability [1,2,3]. Among these components, the herbaceous layer plays an irreplaceable ecological role within vegetation communities [4]. It becomes a crucial link in the sustainable development of ecosystems through processes such as soil and water conservation, provision of biological habitats, and facilitation of material cycling and energy flow [5,6]. However, different types of overstory vegetation (such as forests, shrublands, grasslands) significantly influence resource allocation and ecological processes in the underlying herbaceous layer [7,8]. Various vegetation types create differentiated microhabitats through canopy light transmission rates, root competition, and litter input [9]; this differentiation subsequently regulates the diversity maintenance, spatial distribution patterns, and productivity of herbaceous plants [10,11]. Understanding this hierarchical regulatory mechanism is essential for revealing ecological succession patterns, assessing habitat quality, and formulating biodiversity conservation strategies—holding significant theoretical and practical implications.

Traditional studies often simplify the relationship between biomass and biodiversity in herbaceous layers into linear models, neglecting the nonlinear regulatory effects of spatial scale factors and habitat heterogeneity gradients on ecological patterns [12]. Fixed-area sampling methods may underestimate or overestimate complex community characteristics, while conventional diversity indices struggle to capture the scale-dependent nature of spatial variation. The power law model was originally formulated based on species occurrence frequency and density [13]. Chen et al. (2019) further validated its applicability to a range of vegetation measurement indicators, such as coverage, biomass, density, and frequency [14]. Its application has been extensively documented across diverse ecosystems, including intertidal macroalgal communities [15], grasslands [16], and desert environments [17]. Moreover, the model has been effectively integrated with remote sensing technology to develop early warning systems for ecological restoration [17,18], as well as to identify key species involved in the restoration of degraded ecosystems [19]. The core objective is to reveal how the distribution or spatial association of vegetation attributes changes with varying scales, typically manifesting as a linear relationship on double logarithmic coordinates. This approach simplifies complex systems and provides a framework for cross-scale predictions.

The minimum sampling area is defined as the smallest plot size that adequately represents the species composition and ecological characteristics of a plant community [20,21]. The quantitative determination of the minimum area is typically based on species–area relationships (SARs), which serve as one of the foundational models in ecology and describes the general pattern that species richness increases with the expansion of the sampled area. This relationship is commonly represented using a power function model [22,23]. This model plays a crucial role in elucidating the mechanisms of community construction, the effects of habitat fragmentation, and the principles of protected area design [24,25]. The species–area relationship (SAR) is primarily shaped by two distinct ecological mechanisms. The first, known as the habitat heterogeneity hypothesis, suggests that larger areas encompass a greater diversity of microhabitats, which in turn support higher levels of species richness [26]. The second mechanism is the habitat fragmentation effect, whereby both the magnitude of habitat loss and the spatial configuration of fragmented landscapes jointly influence SAR dynamics. Evidence indicates that under conditions of low habitat loss, moderate fragmentation may create heterogeneous environments that facilitate species coexistence. In contrast, when habitat loss is substantial, fragmentation tends to increase isolation and reduce habitat availability, thereby intensifying the risk of local extinctions [25]. Furthermore, SAR parameters exhibit notable scale dependence: at smaller scales, local processes such as diffusion constraints predominantly influence patterns, whereas larger scales reflect overarching regional species distribution trends [23]. The implementation of a nested sampling design has been demonstrated to effectively capture this scale-dependent characteristic [20].

This study innovatively combines the concepts of minimum area and power law model analysis to investigate how herbaceous community minimum area responds to changes in overstory vegetation structure under different canopy types in the agro-pastoral ecotone of northern China. Additionally, it aims to quantify the relationship between diversity and heterogeneity through the analysis of power law analyses. Furthermore, we explore whether microhabitat heterogeneity regulates the nonlinear transition of the biomass–diversity relationship through scale effects. By integrating observations with model analyses, this research will provide a theoretical basis for revealing mechanisms of vegetation layer interactions and optimizing ecosystem management.

## 2. Materials and Methods

### 2.1. Overview of the Study Site

The investigation was conducted in Youyu County, Shanxi Province (N 39°59′, E 112°19′), China, with an average altitude of 1348 m, an average annual temperature of 4.7 °C, the coldest month (January) average temperature of −14 °C (−17.8 °C to −9.9 °C), the hottest month (July) average temperature of 20.5 °C (18.9 °C to 22.4 °C), and a total accumulated temperature of ≥0 °C of 2600 to 3600 d °C. The first frost period is early- to mid-September, with a frost-free period of 100 to 120 days. The annual total solar radiation is 598 KJ/cm^2^, with an annual sunshine duration of 2600 to 2700 h; the annual rainfall is 435 mm, belonging to the temperate continental monsoon climate.

Youyu is a renowned benchmark for ecological restoration in arid and semi-arid areas of China, having engaged in afforestation and greening efforts for over 70 years. The primary afforestation plant species include small-leaf poplar (*Populus simonii* Carrière), caragana (*Caragana korshinskii* Kom.), and pine (*Pinus sylvestris var. mongholica* Litv.). The grassland type in this area is the temperate mountain grassland of the *Leymus*-*Puccinellia tenuiflora* miscellaneous grass community type, which is adjacent to the Inner Mongolia Plateau grassland area. The soil is calcareous brown soil with a pH of 9.2, which is alkaline.

### 2.2. Experimental Methods

**Figure 1:**
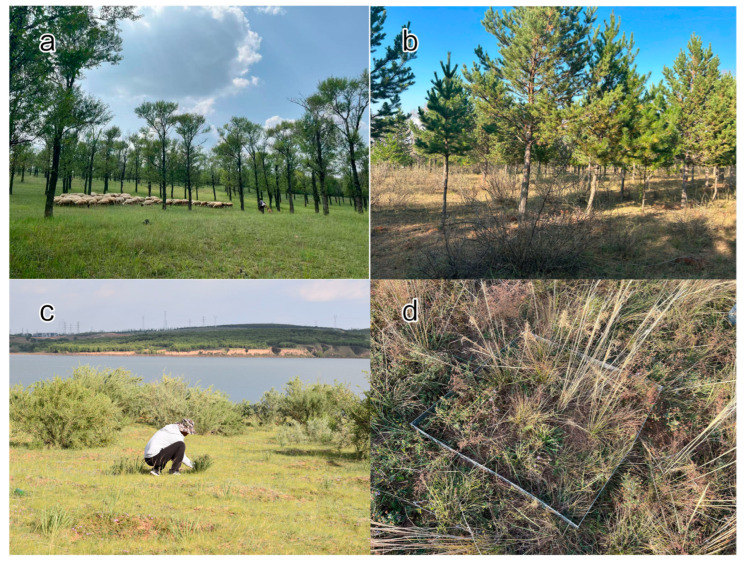
Photographs of the sample plots. Note: (**a**) *Populus simonii* Carrière community (SP); (**b**) *Pinus sylvestris var. mongholica* Litv. and *Caragana korshinskii* Kom. mixed community (PC); (**c**) *Caragana korshinskii* Kom community (RC); and (**d**) saline grassland community (SG).

In the field investigation, three representative native tree and shrub vegetation types, as well as the herbaceous layer beneath typical saline-alkali grasslands, were selected as research subjects. There are four types of sample plots used in the research. Small-leaf poplar plot (SP) is broad-leaved forests primarily composed of *Populus simonii* Carrière. The average tree height of SP is 15 to 20 m, the plant spacing is about 6 m, and it is a sparse forest (Figure 1a). Pine and caragana mixed plot (PC) mainly comprises *Pinus sylvestris var. mongholica* Litv. and *Caragana korshinskii* Kom. In the plantation, pine trees and caragana are interplanted in an alternating pattern. The spacing between pine trees is maintained at 3 m, while the spacing between caragana shrubs is 2 m, with a row spacing of 3 m. Both pine and caragana reach an approximate height of 2 m (Figure 1b). Row-seeded caragana plot (RC) mainly comprises *Caragana korshinskii* Kom. The spacing between its plants and rows is both 2 m (Figure 1c). The saline grassland plot (SG) exhibits severe soil salinization, with surface soil pH (0–10 cm depth) ranging from 9 to 10. The concentrations of exchangeable cations, including potassium (K^+^), calcium (Ca^2+^), sodium (Na^+^), and magnesium (Mg^2+^), are measured at 90 ± 5 mg·kg^−1^, 1400 ± 50 mg·kg^−1^, 400 ± 20 mg·kg^−1^, and 300 ± 18 mg·kg^−1^, respectively. Saline grasslands are characterized solely by herbaceous vegetation, which is predominantly composed of *Leymus secalinus* (Georgi) Tzvelev, which contributes to over 80% of the aboveground biomass. Associated species include salt-tolerant plants such as *Puccinellia distans* (Jacq.) Parl., *Artemisia anethifolia* Stechm., and *Potentilla anserina* L. (Figure 1d). The grassland, low caragana shrub, as well as herbaceous layer under the forest are mainly used for sheep grazing.

**Figure 2:**
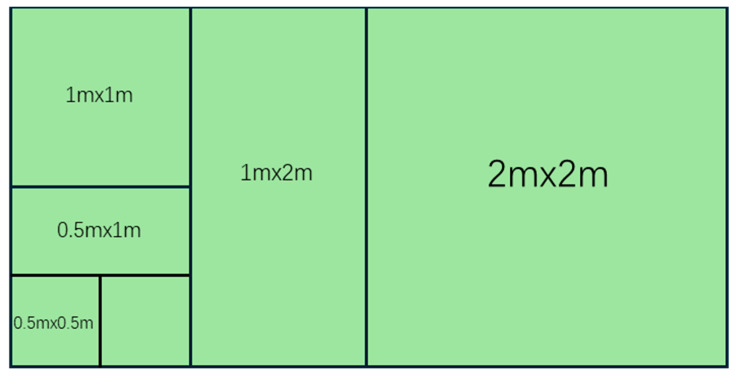
The nested sampling procedure from 0.25 m^2^ to 8 m^2^.

In August of 2024, we conducted a field vegetation survey. For the four types of planting covers (SP, PC, RC, SG), three parallel sample strips are set up for each type. The direction of the sample strips is determined based on the terrain and vegetation gradient to ensure the variation of the coverage of the micro-environment. Five nested sample square sequences are arranged on each sample strip. The sample square product starts from 0.25 m^2^ and expands step-by-step (0.25 → 0.5 → 1 → 2 → 4 → 8 m^2^), forming a spatially continuous gradient sequence. The sequence spacing of adjacent nested sample squares within the same band should be ≥10 m to avoid overlapping. The spacing between different bands is greater than 50 m to ensure independence. There were a total of 15 nested sequences (3 sample bands ×5 sequences) for each vegetation type, with a total of 60 sampling units covering heterogeneous habitats. Within each area level, we recorded the species names, abundance, coverage, and height of all vascular plants. The 8 square meters of sample plots were simultaneously and evenly harvested and weighed to determine the aboveground biomass. The design of the sample zone considers both the canopy gradient and the soil transition zone (Figure 2).

#### 2.2.1. Species–Area Relationship Analysis

The nested sampling method was employed, starting with 0.25 m^2^ and progressively expanding the plot size (0.25 → 0.5 → 1 → 2 → 4 → 8 m^2^). The plant species observed within each plot were recorded. Species–area relationship fits the Power Curve below:

*S* = *c* × *A^z^* (or the logarithmic expression: *lg*(*S*) = *lg*(*c*) + *z*·*lg*(*A*)) (1)

In the above formula, *S* is the number of plant species observed within each plot (Species Richness), *A* is the plot size (Area), and *c* and *z* are constants. *c* represents the expected number of species per unit area (*A* = 1 × m^2^). *z* reflects the sensitivity of species richness to increases in area. A higher *z*-value (approximately 0.25–0.55) indicates highly fragmented habitats and a faster rate of species accumulation in response to area expansion, and implies large-scale protection is required. In contrast, low *z* values (approximately 0.1–0.2) imply that broad-scale area protection may be less critical, with greater emphasis required on the management of specific microhabitats.

Although alternative models such as the logarithmic SAR model have been proposed by Rosenzweig and Dengler [27,28], the power law model is widely used in vegetation ecology due to its robustness across scales and interpretability of the z parameter [14]. Our preliminary analyses indicated that the power law model provided a better fit to the data than the logarithmic model. The power law model was fitted by linearizing it through logarithmic expression: *lg*(*S*) = *lg*(*c*) + *z* × *lg*(*A*). The parameters were estimated using ordinary least squares (OLS) regression, and the assumptions of linearity, normality of residuals, and homoscedasticity were verified through visual inspection of residual plots and the Shapiro–Wilk test for normality.

#### 2.2.2. Taylor’s Power Law Model Principle

Taylor’s power law posits that there is a power law relationship between the population density (*m*) and variance (*V*) of species in nature:

*V* = *a* × *m^b^* (2)

In the above formula, *a* is a parameter related to the sampling scheme; *b* is the aggregation index, indicating the level of spatial aggregation of the population. *b* = 1 indicates that the population type is non-density-limited aggregation; *b* < 1 indicates that the function is a decreasing function, indicating that the population type is density-limited aggregation, and vice versa, *b* > 1 indicates that the population type is inverse density-limited aggregation.

Shiyomi (2001) combined the “binary occurrence frequency” survey with Taylor’s power law model analysis to quantitatively describe the spatial heterogeneity of grassland communities [29]. The population density of species *i* obtained from the “binary occurrence frequency” survey is represented by the occurrence frequency *p_i_*, and since *p_i_* follows a binomial distribution, its variance is *p_i_*(1 − *p_i_*)/*n*. If the variance of the number of occurrences of species *i* in the large quadrat is represented by *v_i_*, then *v_i_*/*n*^2^ represents the variance of the actual observed frequency of species *i*. Therefore, using the Taylor’s power law model analysis for a community with *s* plant species, the above two variances have the following relationship:

*v_i_*/*n*^2^ = *a* × *p_i_^b^*, *i* = 1, 2 …, *s* (3)

where *s* is the number of species appearing in the community, *p_i_* is the occurrence frequency of species *i*, and *a* and *b* are constants, representing random variables.

Taking the logarithm of the two variances, let *y_i_ = ln(v_i_*/*n*^2^) and *x_i_* = *ln*[*p_i_*(1 − *p_i_*)/*n*]. Theoretically, there is a linear relationship between *y_i_* and *x_i_*:

*y_i_* = *b* × *x_i_* + *ε_i_*, *i* = 1, 2 …, *s* (4)

The regression line of Taylor’s power law model represents the average level of spatial heterogeneity of the community. Therefore, in the above formula, *ε_i_* is the difference in spatial heterogeneity between species *i* and the community level *δ_c_*.

If the distance *δ_i_* between the *y_i_* value of species *i* and the reference line *y* = *x* along the *y*-axis can represent the degree of spatial heterogeneity of species *i*, then

*δ_i_* = *b* × *x_i_* + *ε_i_* − *x_i_*, *i* = 1, 2 …, *s* (5)

When *δ_i_* = 0, species *i* is randomly distributed; when *δ_i_* > 0, species *i* has a more heterogeneous spatial distribution than random distribution, that is, concentrated distribution; when *δ_i_* < 0, species *i* has a more uniform distribution than random distribution, that is, regular distribution.

The community heterogeneity index $\delta_{c}$ was calculated as the abundance-weighted average of species-specific *δ_i_* values:

(6) $$\delta_{c}=\Sigma (p_{i}\times \delta_{i}),i=1,2\ldots ,s$$

where *p_i_* and *δ_i_* are the occurrence frequency and heterogeneity index of species *i*, respectively. The larger the *δ_c_* value, the higher the degree of overall spatial distribution heterogeneity of the community, and the judgment basis for the degree of spatial distribution heterogeneity is the same as the *δ_i_* value.

To evaluate community heterogeneity, Ma (2015) further proposed the concept of community aggregation critical diversity (*CACD*) based on the parameters *a* and *b* in the power law, which is the critical value between aggregative communities and uniform communities [30]:

(7) $$CACD\;=\;a{\;\times \;b}^{1-b}$$

The Shannon–Wiener diversity index *H*′ is calculated as follows:

*H*′ = −Σ(*p_i_*′ × *ln*(*p_i_*′)), *i* = 1, 2 …, *s* (8)

where *p_i_*′ is calculated by the following formula:

*p_i_*′ = (*p_i_* × (1 − *p_i_*)/*n*)*^b^*, *i* = 1, 2 …, *s* (9)

Pielou’s evenness index *J*′ is calculated as follows:

*J*′ = *H*′/*H_max_*, *i* = 1, 2 …, *s* (10)

where *H_max_* is the maximum value of *H*′.

The dominance index *D* is calculated as follows:

*D* = Σ(*p_i_^2^*), *i* = 1, 2 …, *s* (11)

### 2.3. Statistical Analysis

Microsoft Excel 2016 and SAS V8.2 were used for data organization and calculation and single-factor variance analysis. The significance of differences between treatments was determined by Duncan’s method for multiple comparisons (*p* < 0.05); SigmaPlot 14.0 was used for plotting.

## 3. Results

### 3.1. Species Composition and Distribution Patterns Across Vegetation Types

The vegetation community is dominated by temperate perennial species, totaling 33 species (Table A1). Poaceae and Asteraceae emerge as the most dominant families, each represented by six species, followed by Fabaceae with four species; the remainder comprises forbs. Across different plots, significant differences in species richness are observed: RC plots show the highest richness (25 species, 75.8%), while SG plots exhibit the lowest (12 species, 36.4%). SP and PC contain 23 and 19 species, respectively. Species-sharing patterns reveal that SP and RC plots demonstrate higher numbers of shared species, whereas SG plots demonstrate both the fewest shared species and the highest endemism (three unique species, representing 25% of its total species). This suggests SG may constitute a potential specialized habitat within salt-tolerant plant communities.

**Figure 3:**
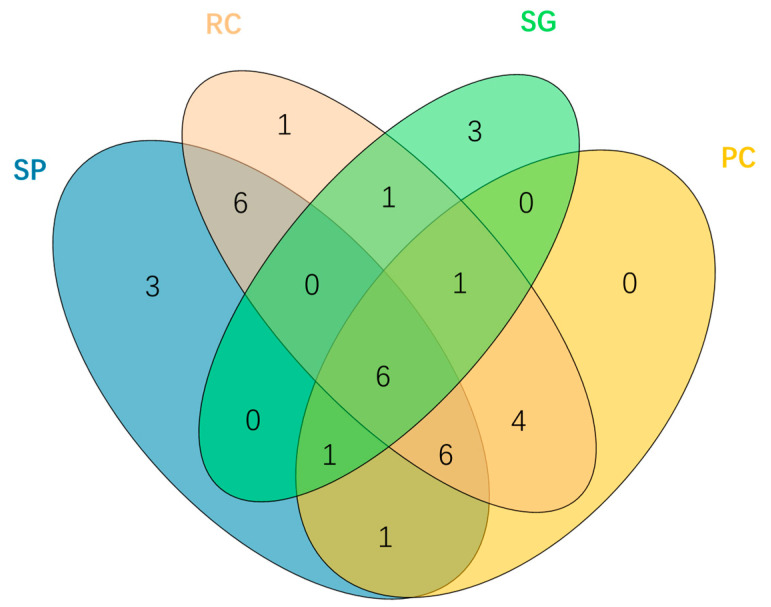
Venn diagram of species occurring in the investigated plots. Note: 33 species are distributed across the herbaceous layers of four distinct vegetation types (SP, PC, RC, SG). The numbers in the diagram represent the count of species that occurred in the corresponding type. The overlapping regions among the ellipses illustrate the shared species between these vegetation types, while the central intersection reflects the species common to all four types.

The six overlapping species in the center of the Venn diagram are present in all plot types and constitute foundational elements within this ecosystem (Figure 3). Among these, *Leymus chinensis* (Trin.) Tzvelev—an advantageous forage in this study area—exhibits its highest biomass in RC plots (65 g/m^2^), indicating its ecological superiority and suggesting that RC may serve as a biodiversity hotspot. From an endemic perspective, both SP and SG each harbor three unique species; notably, unique taxa comprise a significant proportion within SG at 25% of its total number of recorded species. In contrast to this finding, SG has been observed to share the fewest common taxa with other plot types among salt-tolerant plant communities.

### 3.2. Species–Area Relationships of Herbaceous Layer

**Figure 4:**
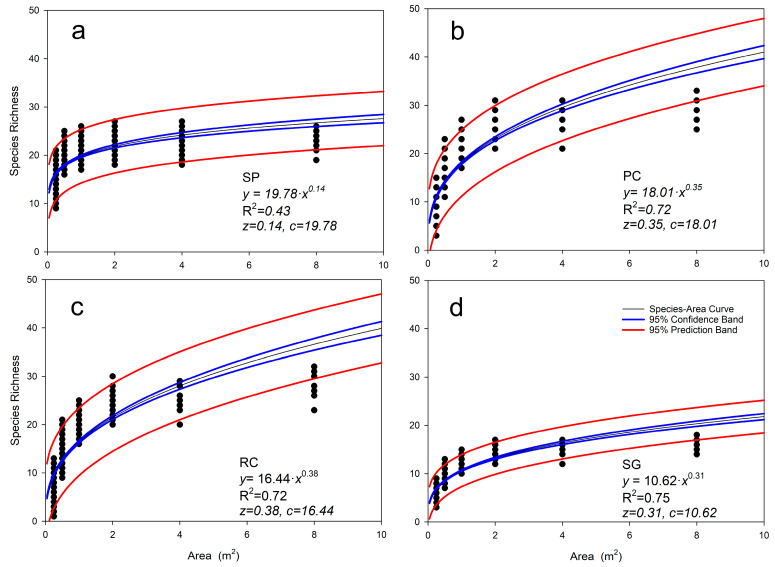
Species–area curves under different vegetation types. (**a**) Species–area curves under SP (R^2^ = 0.43), (**b**) Species–area curves under PC (R^2^ = 0.72), (**c**) Species–area curves under RC (R^2^ = 0.72), (**d**) Species–area curves under SG (R^2^ = 0.75). Small-leaf poplar forest (SP) exhibits minimal area effect (lowest z = 0.14), while saline grassland (SG) shows highest area sensitivity.

Figure 4 illustrates the species–area relationship (SAR) curves derived using the nested pattern method across vegetation types. The *x*-axis denotes the sampling area, while the *y*-axis indicates species richness. It is evident that species richness increases monotonically with the expansion of the sampling area, exhibiting well-fitted power law relationship between species richness (S) and area (A) (*p* < 0.05). A comparison across vegetation types reveals that the SP herbaceous layer has the lowest *z* value (0.14), indicating a minimal area effect. This vegetation type exhibits the highest (*c* = 19.78) species density per unit area (nearly 20 species), yet area explains only a proportion of the variation in species richness. Consequently, expanding the survey area results in minimal gains in species diversity, suggesting that it represents a homogeneous and mature community. Therefore, the conservation priority for the SP area is relatively low, and efforts should focus on microhabitat management.

In contrast, the herbaceous layers of PC and RC demonstrate high sensitivity to changes in area, with relatively high species density. Although the SG herbaceous patches in saline grasslands exhibit low species richness in small areas, the area has the highest explanatory power for species richness variation.

### 3.3. Community Spatial Heterogeneity Parameters

**Figure 5:**
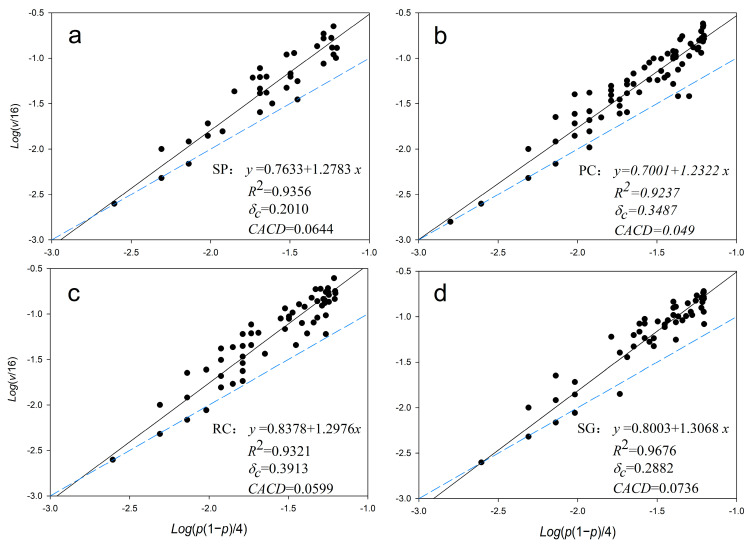
Regression line between species’ occurrence frequency and variance using power-law. (**a**) Regression line of SP, (**b**) Regression line of PC, (**c**) Regression line of RC, (**d**) Regression line of SG. The black solid line is the regression line of Taylor’s power law model for all plant species in the quadrat, the blue dashed lines is line y = x used as a reference.

There is a significant regression relationship between species occurrence frequency and variance (Figure 5), and the determination coefficients are all above 0.92, indicating that the spatial distribution of plant species fits well with Taylor’s power law model, which is suitable for evaluating community spatial characteristics. In the figure, the regression line (solid line) is the regression line of Taylor’s power law model for all plant species in the quadrat, representing the average level of spatial heterogeneity of the community.

If the regression line is used as a reference, more than 44% of the species are above the line, with a regression residual *ε_i_* > 0, indicating that the heterogeneity of these plant species is higher than the community level *δ_c_*, suggesting that the occurrence of these plant species promotes the overall spatial heterogeneity of the community; conversely, plant species located below the line reduce the overall spatial heterogeneity of the community. The dominant species *Leymus secalinus* (Georgi) Tzvelev is always below the regression line (*ε_i_* < 0), and its species spatial heterogeneity is lower than the average level of the community, which remains unchanged regardless of variations in the upper story.

Spatial heterogeneity peaked in caragana shrublands (RC: 0.3913) and pine–caragana mixed forests (PC: 0.3487), but was minimal in small-leaf poplar forests (SP: 0.20). High *δ_c_* values reflect microenvironmental diversification through shrub root networks and litter heterogeneity.

The blue dashed line *y* = *x* is used as a reference. Comparing the position relationship between the scatter points and the reference line, it is found that more than 82.5% of the points are above the line *y* = *x*; that is, *δ_i_* > 0, indicating that the most of the herb layer is concentrated; on the contrary, some points are below the line *y* = *x*, *δ_i_* < 0, showing a regular distribution. In addition, under SP and SG, the scatter points representing *Leymus secalinus* (Georgi) Tzvelev are below the line *y* = *x*, showing a regular distribution (*δ_i_* < 0); while under PC and RC, they are above the line *y* = *x*, showing a concentrated distribution (heterogeneous distribution).

With the slope coefficient of the regression line, parameter *b* > 1, the degree of population aggregation increases as a function of population density, indicating the presence of a positive feedback mechanism between these two factors. This mechanism is analogous to the inverse density-dependent effect observed in population dynamics. Consequently, such populations are referred to as exhibiting aggregation-degree inverse density dependence. The observed aggregation behavior in these populations may be attributed to the inherent aggregative tendencies of individual species.

By analyzing the changes in the community heterogeneity index *δ_c_* and the community aggregation critical diversity (*CACD* values) under different treatments, it can be seen that the community heterogeneity of the SP grassland is the lowest, and the *CACD* is at a medium level, and the *CACD* reaches its maximum under SG; the overall heterogeneity index value of the community under RC treatment is the largest, and the community aggregation critical diversity decreases.

### 3.4. Community Spatial Diversity Parameters

**Figure 6:**
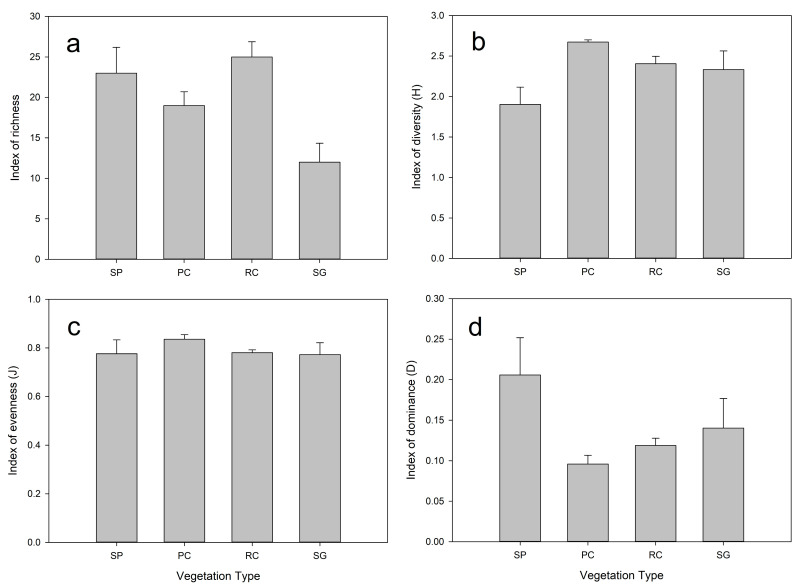
Community diversity index based on Taylor’s power law. (**a**) Index of richness of four community, (**b**) Index of diversity of four community, (**c**) Index of evenness of four community, (**d**) Index of dominance of four community. Note: PC optimizes diversity with minimal dominance, while SP shows high dominance and low diversity.

Plant diversity exhibited pronounced variations across vegetation types, reflecting distinct ecological trade-offs mediated by canopy structure (Figure 6). The Shannon–Wiener diversity index (*H*′) peaked in pine–caragana mixed forests (PC: *H*′ = 2.67 ± 0.03), significantly exceeding values in small-leaf poplar forests, caragana shrublands (RC:), and saline grasslands (F(3,16) = 3.75, *p* = 0.0413). This trend aligned with Pielou’s evenness (*J*′), where PC achieved a potential higher evenness (F(3,16) = 0.58, *p* = 0.636), indicative of balanced species co-occurrence under moderated canopy complexity.

Conversely, SP exhibited the highest dominance index (SP: *D* = 0.21 ± 0.05; *p* < 0.05), signifying strong competitive exclusion by few species (e.g., *Leymus secalinus* (Georgi) Tzvelev). Despite RC supporting the highest species richness (25 species), its diversity metrics (RC: *H*′ = 2.40 ± 0.09; *J*′ = 0.78 ± 0.01) were intermediate, reflecting moderate dominance (RC: *D* = 0.11 ± 0.03). SG showed the lowest richness (12 species) coupled with high dominance (SG: *D* = 0.14 ± 0.04), consistent with abiotic stress filtering species composition.

Notably, our results highlight a trade-off between biodiversity and heterogeneity: PC optimized biodiversity metrics, including species diversity (*H*′) and evenness (*J*′), while minimizing dominance (*D*), suggesting more balanced resource partitioning among species. RC emphasized spatial heterogeneity (*δ_c_*) as well as species richness, thereby functioning as a biodiversity hotspot. SP exhibited high dominance and low diversity, indicative of competitive exclusion in structurally homogeneous canopies. The low species richness and high community-aggregated competitive dominance (*CACD*) of SG highlight its susceptibility to abiotic degradation.

## 4. Discussion

### 4.1. Synthesis of Key Innovations: The Integration of the Minimum Area Quantification and the Power Law Model Unravel Vegetation Dynamics

Our study pioneers the integration of minimum area quantification with Taylor’s power law framework, providing a robust tool to decode scale-dependent ecological patterns in agro-pastoral grasslands. This methodological synergy revealed three pivotal advances. First, scale-driven heterogeneity gradients: The power law parameter *z*, derived from species–area relationships (SARs), served as a diagnostic indicator for habitat fragmentation [31,32]. The significantly low *z* value in herbaceous layers of small-leaf poplar forests (SP, *z* = 0.14) indicates a homogeneous, stable community where species richness saturates rapidly with minimal area expansion. Conversely, the high *z* in saline grasslands (SGs) reflects high habitat fragmentation, necessitating larger protected areas to capture microhabitat endemic species [25,26], but now contextualized within canopy-mediated microhabitats. It is noteworthy that the *z* value of PC (mixed forest) falls between the two extremes while exhibiting the highest species diversity. This indicates that moderate canopy complexity enhances spatial heterogeneity by generating diverse gradients of light, water, and nutrients (e.g., forest windows and shrub interzones), thereby providing empirical support for the theory that moderate disturbance promotes diversity [2].

Second, canopy-dependent shifts in dominant species spatial strategy [33,34]: This study demonstrated that 82.5% of the herbaceous species displayed aggregated distribution patterns (δi > 0), with the dominant species *Leymus secalinus* (Georgi) Tzvelev shifting from a homogeneous (δi < 0) to an aggregated spatial arrangement under PC/RC conditions. This observed pattern can be attributed to the interplay between habitat fragmentation and total vegetation volume. In SP/SG communities, where canopy coverage is relatively low, environmental filtering processes—such as light limitation in SP and salinity stress in SG—predominantly influence community assembly, leading to the widespread but uniform dispersal of a limited set of stress-tolerant species. Conversely, the heterogeneous canopy structure of PC/RC generates physical barriers (e.g., shrub basal stems), which partition the habitat into discrete “micro-island” units, thereby facilitating species aggregation [33]. These results are consistent with the “small-scale agglomeration-driven SAR” mechanism documented in tropical forests [35]. Importantly, this study extends previous understanding by elucidating how canopy configuration regulates dispersal limitations—particularly, the shrub within PC mixed forests may function as a relay station for animal-mediated seed dispersal, thereby mitigating the dispersal constraints faced by herbaceous species [36].

Additionally, optimal vegetation configuration for biodiversity–heterogeneity trade-offs: PC exhibited the highest diversity (*H*’) and lowest dominance (*D*), signifying balanced resource partitioning in mixed-canopy systems. RC achieved the peak spatial heterogeneity (*δ_c_*), highlighting shrubs as engineers of microenvironmental variability. These results demonstrate that vegetation types differentially mediate the biodiversity–heterogeneity nexus, with PC optimizing diversity, while RC maximizes spatial complexity.

### 4.2. Ecological Implications in Theoretical Context: Convergence and Divergence with Existing Paradigms

Our power law-derived indices transcend descriptive statistics to reveal mechanistic drivers of community assembly. The pervasive species aggregation (*δ_c_* > 0) observed across vegetation type, particularly in RC (*δ_c_* = 0.38) and PC (*δ_c_* = 0.32), signifies that biotic interactions (competition or facilitation) override stochastic processes in structuring herbaceous communities. This aligns with Shiyomi’s framework, where aggregated distributions in grasslands reflect niche partitioning [29]. Crucially, >82.5% of species exhibited *δ_i_* > 0, reinforcing that spatial clustering is a universal strategy for resource acquisition in heterogeneous environments, as posited by Tilman’s resource competition theory [37]. The aggregated pattern was consistent with multiple findings in arid or semiarid natural grasslands in Loess Plateau and elsewhere [16,32,38,39].

The peak *CACD* value in SG acts as an early-warning signal for ecosystem transition. Ma [30] originally proposed *CACD* to distinguish aggregative vs. uniform communities, but our data extend its utility: high *CACD* under saline stress indicates proximity to a critical threshold where abiotic filters (salinity) drive a phase shift from uniform to aggregated states—a pattern mirroring degradation thresholds in arid grasslands [40]. The maximized diversity and minimized dominance were observed in PC mixed forests mainly comprising pine and caragana, making it a biodiversity hotspot. Moderate canopy complexity in mixed forests reduces light competition while enhancing niche dimensionality—enabling coexistence of shade-tolerant forbs and light-demanding grasses [41,42].

As for power law validity and diversity mechanisms, the high model fit for Taylor’s regression validates its efficacy in quantifying spatial heterogeneity, consistent with Shiyomi [29] and Song [32]. This confirms the universality of power law scaling for diagnosing vegetation spatial patterns across biomes. However, our findings may challenge conventional recognition. *Leymus secalinus* (Georgi) Tzvelev, the dominant species of the studied area, shifted from uniform (SP/SG) to aggregated (PC/RC) distributions, contradicting grazing-centric models, which attributed *Leymus secalinus* (Georgi) Tzvelev aggregation solely to livestock pressure [36]. Our data implied shade in SP and salinity in SG suppress clonal integration of *Leymus secalinus* (Georgi) Tzvelev, while shrub-facilitated microsites in RC release its innate clonality. It is suggested that nonlinear canopy–soil interactions override linear models. Similarly, high richness always necessitates high heterogeneity, but low diversity (*H′* = 1.9) is associated with high uniformity (*δ_c_* = 0.18) in the community of SP. In closed canopies of SP, competitive exclusion trumps niche homogenization in the understory herbaceous layer, negating assumed diversity–heterogeneity coupling [41,43]. This highlights that power law parameters are not just statistics but mechanistic lenses for dissecting ecosystem stability thresholds and assembly rules.

### 4.3. Practical Implications: Optimizing Canopy Structures for Sustainability

The significantly higher Shannon diversity (*H′*) and lower dominance (*D*) observed in pine–caragana mixed forests (PC) underscore the ecological advantage of moderately open canopies (~50% canopy openness). This structure balances light penetration and resource partitioning, facilitating coexistence of shade-tolerant forbs and light-demanding grasses (e.g., *Leymus secalinus* (Georgi) Tzvelev). To enhance biodiversity in the agro-pastoral ecotone, we advocate replacing homogeneous small-leaf poplar monocultures (SPs) with pine–caragana mixed plantations. This strategy aligns with our finding that PC supports 19 species with minimal dominance (Figure 5), promoting resilient grassland communities under canopy mediation [44].

Saline grasslands (SGs) exhibit critically high community aggregation critical diversity (CACD), signaling proximity to an ecological threshold where salinization stress overrides biotic interactions. To reverse degradation, introducing caragana shrub (RC) patches is recommended. RC’s peak spatial heterogeneity (*δ_c_* = 0.38) demonstrates its capacity to engineer microhabitats: shrubs reduce topsoil salinity via root exudates and litter input while elevating microenvironmental heterogeneity(*δ_c_*) [45]. This approach mirrors successful restoration paradigms in semi-arid grasslands, where shrub integration lowered degradation thresholds by 40–60%. This specifies the positive impact of shrub on the herbaceous layer and is consistent with a preceding report [46]. Song et al. (2005) proposed that canopy represents another heterogeneous factor influencing vegetation community structure and dynamics, as it regulates soil moisture patterns that shape plant distribution and diversity. This perspective is further corroborated by the results of Moeslund (2013) and Deng (2020) in arid or semi-arid regions [32,42,46].

Power law parameters offer actionable metrics for adaptive grassland management. Low *z* zones (e.g., SP) indicate homogeneous communities where species richness saturates rapidly. Management should prioritize microhabitat interventions (e.g., selective thinning, understory enrichment) rather than large-scale protection [47]. High *z* zones (e.g., SG) reflect high habitat fragmentation requiring landscape-scale conservation (e.g., corridors, protected areas ≥ 8 m^2^) to capture area-sensitive diversity. High *δ_c_* clusters (e.g., RC) serve as biodiversity hotspots warranting targeted preservation. We propose integrating *z* and *δ_c_* into regional ecological monitoring frameworks to dynamically diagnose grassland health and guide management investments.

## 5. Conclusions

This study demonstrates that integrating Taylor’s power law with species–area relationship analysis effectively captures vegetation characteristics in the Loess Plateau’s agro-pastoral ecotone. Our results show that canopy structure drives spatial strategies. *Leymus secalinus* (Georgi) Tzvelev shifts from uniform under closed canopies (SP) or saline stress (SG) to aggregated in mixed forests (PC) and shrublands (RC), indicating abiotic filters override biotic interactions in stressful habitats. Pine–caragana mixed forests optimize biodiversity, and PC achieves the highest Shannon diversity (*H*′) and lowest dominance (*D*), attributed to moderated light regimes and niche partitioning. Shrublands enhance heterogeneity, and RC exhibits peak spatial variability, positioning shrubs as keystone engineers for microenvironment complexity. Maximized *CACD* in SG acts as an early-warning threshold for ecosystem transition. These insights advocate for mixed-canopy afforestation and shrub-mediated restoration as sustainable strategies to balance biodiversity and spatial heterogeneity in ecologically vulnerable regions.

## Data Availability

The data presented in this study are available on request from the corresponding author. The data are not publicly available due to privacy and ethical restrictions.

## Abbreviations

The following abbreviations are used in this manuscript:

SP	Small-leaf poplar forest
PC	Pine–caragana mixed forest
RC	Row-seeded caragana shrubland
SG	Linear dichroism saline grassland
SAR	Species–area relationship
CACD	Community aggregation critical diversity

## Appendix A

**Table A1:** Plants occurring in the sampling plot.

Species Name	Functional Group	Life Form	Aboveground Biomass (g/m^2^)			
			SP	PC	RC	SG
*Agropyron cristatum* (L.) Gaertn.	Grass	Perennial herb	-	35 ± 5.45	23 ± 3.65	8.8 ± 1.29
*Allium chrysanthum* Regel	Forb	Perennial herb	-	10 ± 1.7	10 ± 1.81	-
*Artemisia brachyloba* Franch.	Forb	Perennial herb	42 ± 2.8	-	15 ± 2.33	-
*Artemisia frigida* Willd.	Forb	Perennial herb	15 ± 1.98	20 ± 3.2	28 ± 4.4	-
*Aster altaicus* Willd.	Forb	Annual herb	-	25 ± 3.95	12 ± 1.95	-
*Astragalus melilotoides* Pall.	Legume	Perennial herb	-	28 ± 4.4	18 ± 3.13	-
*Carex duriuscula* subsp. *rigescens* (Franch) S.Y.Liang et Y.C.Tang	Forb	Perennial herb	65 ± 3.5	40 ± 6.2	35 ± 5.45	12 ± 2
*Cleistogenes squarrosa* (Trin.) Keng	Grass	Perennial herb	39 ± 3.6	46 ± 7.1	50 ± 8.2	-
*Cynanchum thesioides* (Freyn) K. Schum	Forb	Perennial herb	11 ± 1.58	-	15 ± 2.45	-
*Festuca ovina* L.	Grass	Perennial herb	51 ± 2.6	60 ± 7.6	55 ± 8.45	9.8 ± 1.55
*Gentiana scabra* Bunge	Forb	Perennial herb	11 ± 1.99	-	18 ± 2.9	-
*Hippophae rhamnoides* L.	Shrub	Shrub	46 ± 2.6	-	-	-
*Inula japonica* Thunb.	Forb	Perennial herb	20 ± 2.5	40 ± 5.12	50 ± 7.7	8.2 ± 1.7
*Ixeridium sonchifolium* (Maxim.) Shih	Forb	Annual herb	-	-	12 ± 2.16	20 ± 3.2
*Lespedeza bicolor* Turcz.	Legume	Shrub	12 ± 1.95	36 ± 5.6	40 ± 6.2	-
*Leymus chinensis* (Trin. ex Bunge) Tzvelev	Grass	Perennial herb	45 ± 3.5	52 ± 8	65 ± 9.95	13 ± 2.15
*Leymus secalinus* (Georgi) Tzvelev	Grass	Perennial herb	40 ± 4	42 ± 6.5	20 ± 3.2	10 ± 1.59
*Medicago ruthenica* (L.) Trautv.	Legume	Perennial herb	30.5 ± 3.1	42 ± 5.9	-	-
*Medicago sativa* L.	Legume	Perennial herb	38 ± 3.3	12 ± 2	15 ± 2.79	-
*Oxytropis caerulea* (Pallas) Candolle	Legume	Perennial herb	8.6 ± 1.47	-	-	-
*Parthenocissus chinensis* C. L. Li	Forb	Annual herb	12 ± 2.6	-	8 ± 1.4	-
*Potentilla acaulis* L.	Forb	Perennial herb	-	-	13 ± 2.15	-
*Potentilla supina* L.	Forb	Perennial herb	-	-	-	16 ± 2.6
*Puccinellia distans* (Jacq.) Parl.	Grass	Perennial herb	-	-	-	25 ± 3.95
*Salsola collina* Pall.	Forb	Annual herb	18 ± 2.5	8 ± 1.4	-	17 ± 2.75
*Setaria viridis* (L.) P. Beauv.	Grass	Annual herb	21 ± 4	-	25 ± 3.95	-
*Sibbaldianthe bifurca* (L.) Kurtto & T. Erikss.	Forb	Perennial herb	5 ± 1.21	8 ± 1.35	8 ± 1.2	-
*Stipa capillata* L.	Grass	Perennial herb	42 ± 3.9	60 ± 9.2	62 ± 9.5	-
*Teloxys aristata* (L.) Moq.	Forb	Annual herb	19 ± 2.8	15 ± 2.45	10 ± 1.53	15 ± 2.45
*Tephroseris kirilowii* (Turcz. ex DC.) Holub	Forb	Perennial herb	15 ± 2.71	-	-	-
*Thymus mongolicus* (Ronniger) Ronniger	Shrub	Shrub	-	23.5 ± 3.725	80.1 ± 12.215	-
*Viola yedoensis* Makino	Forb	Perennial herb	10 ± 1.26	-	8 ± 1.3	-
Unverified forb	Forb	Herb	-	-	-	10 ± 1.55

## References

[1] Dietrich P., Ebeling A., Meyer S.T., Asato A.E.B., Bröcher M., Gleixner G., Huang Y., Roscher C., Schmid B., Eisenhauer N. (2024). Plant diversity and community age stabilize ecosystem multifunctionality. Glob. Change Biol..

[2] Hautier Y., Tilman D., Isbell F., Seabloom E.W., Borer E.T., Reich P.B. (2015). Anthropogenic environmental changes affect ecosystem stability via biodiversity. Science.

[3] Eisenhauer N., Hines J., Isbell F., Van Der Plas F., Hobbie S.E., Kazanski C.E., Lehmann A., Liu M., Lochner A., Rillig M.C. (2018). Plant diversity maintains multiple soil functions in future environments. eLife.

[4] Deng J., Fang S., Fang X., Jin Y., Kuang Y., Lin F., Liu J., Ma J., Nie Y., Ouyang S. (2023). Forest understory vegetation study: Current status and future trends. For. Res..

[5] Salemaa M., Hotanen J.P., Oksanen J., Tonteri T., Merilä P. (2023). Broadleaved trees enhance biodiversity of the understorey vegetation in boreal forests. For. Ecol. Manag..

[6] Bump J.K., Webster C.R., Vucetich J.A., Peterson R.O., Shields J.M., Powers M. (2009). DUngulate carcasses perforate ecological filters and create biogeochemical hotspots in forest herbaceous layers allowing trees a competitive advantage. Ecosystems.

[7] Deng T., Du Q., Zhu Y., Queenborough S.A. (2025). Environmental drivers of herbaceous plant diversity in the understory community of a warm-temperate forest. Plant Divers..

[8] Wrońska-Pilarek D., Rymszewicz S., Jagodziński A.M., Gawryś R., Dyderski M.K. (2023). Temperate forest understory vegetation shifts after 40 years of conservation. Sci. Total Environ..

[9] Santi I., Carrari E., De Frenne P., Valerio M., Gasperini C., Cabrucci M., Selvi F. (2024). Impact of coppicing on microclimate and understory vegetation diversity in an ancient Mediterranean oak forest. Sci. Total Environ..

[10] Kartalaei Z.M., Kooch Y., Tilaki G.A.D. (2023). Litter and soil properties under woody and non-woody vegetation types: Implication for ecosystem management in a mountainous semi-arid landscape. J. Environ. Manag..

[11] Lyu Q., Luo Y., Liu S., Zhang Y., Li X., Hou G., Chen G., Zhao K., Fan C., Li X. (2022). Forest gaps alter the soil bacterial community of weeping cypress plantations by modulating the understory plant diversity. Front. Plant Sci..

[12] Anderson M.J., Walsh D.C., Sweatman W.L., Punnett A.J. (2022). Non-linear models of species’ responses to environmental and spatial gradients. Ecol. Lett..

[13] Chen J., Shiyomi M. (2014). Spatial pattern model of herbaceous plant mass at species level. Ecol. Inform..

[14] Chen J., Shiyomi M. (2019). A power law model for analyzing spatial patterns of vegetation abundance in terms of cover, biomass, density, and occurrence: Derivation of a common rule. J. Plant Res..

[15] Li X., Chen J., Li J., Wang K., Wang Z., Zhang S. (2022). Determination of intertidal macroalgae community patterns using the power law model. PLoS ONE.

[16] Guan Q., Chen J., Wei Z., Wang Y., Shiyomi M., Yang Y. (2016). Analyzing the spatial heterogeneity of number of plant individuals in grassland community by using power law model. Ecol. Model..

[17] Lin Y., Han G., Zhao M., Chang S.X. (2010). Spatial vegetation patterns as early signs of desertification: A case study of a desert steppe in Inner Mongolia, China. Landsc. Ecol..

[18] Gibson R., Driscoll D., Macdonald K., Williamson G., Nolan R., Doherty T., Nimmo D., Ritchie E., Tozer M., Tasker L. (2025). Remotely Sensed Fire Heterogeneity and Biomass Recovery Predicts Empirical Biodiversity Responses. Glob. Ecol. Biogeogr..

[19] Mou X.M., Yu Y.W., Li X.G., Degen A.A. (2020). Presence frequency of plant species can predict spatial patterns of the species in small patches on the Qinghai-Tibetan Plateau. Glob. Ecol. Conserv..

[20] He C., Fan F., Qiao X., Zhou Z., Xu H., Li S., Zhu J., Wang S., Tang Z., Fang J. (2024). Sampling origins and directions affect the minimum sampling area in forest plots. J. Veg. Sci..

[21] Navarro-Rosales F., Bell M.B.V. (2022). Woody vegetation within semi-abandoned olive groves: Species-area relationships and minimum area values. Mediterr. Bot..

[22] May R.M., Stumpf M.P. (2000). Ecology. Species-area relations in tropical forests. Science.

[23] Chen B., Jiang J., Zhao X. (2019). Species-Area Relationship and Its Scale-Dependent Effects in Natural Forests of North Eastern China. Forests.

[24] Liu J., MacDonald Z.G., Si X., Wu L., Zeng D., Hu G., Ding P., Yu M. (2022). SLOSS-based inferences in a fragmented landscape depend on fragment area and species-area slope. J. Biogeogr..

[25] Zhang H., Chase J.M., Liao J. (2024). Habitat amount modulates biodiversity responses to fragmentation. Nat. Ecol. Evol..

[26] Yan Y., Jarvie S., Zhang Q., Han P., Liu Q., Zhang S., Liu P. (2023). Habitat heterogeneity determines species richness on small habitat islands in a fragmented landscape. J. Biogeogr..

[27] Rosenzweig M.L. (1995). Species Diversity in Space and Time.

[28] Dengler J. (2009). Which function describes the species–area relationship best? A review and empirical evaluation. J. Biogeogr..

[29] Shiyomi M., Takahashi S., Yoshimura J., Yasuda T., Tsutsumi M., Tsuiki M., Hori Y. (2001). Spatial heterogeneity in a grassland community: Use of power law. Ecol. Res..

[30] Ma Z. (2015). Power law analysis of the human microbiome. Mol. Ecol..

[31] Horník J., Janeček Š., Klimešová J., Doležal J., Janečková P., Jiráská Š., Lanta V. (2012). Species-area curves revisited: The effects of model choice on parameter sensitivity to environmental, community, and individual plant characteristics. Plant Ecol..

[32] Song Z., Huang D., Shiyomi M., Wang Y., Takahashi S., Yoshimichi H., Yamamuru Y., Chen J. (2005). Spatial heterogeneity and variability of a large-scale vegetation community using a power-law model. Tsinghua Sci. Technol..

[33] Brooker R.W., Maestre F.T., Callaway R.M., Lortie C.L., Cavieres L.A., Kunstler G., Liancourt P., TielböRger K., Travis J.M.J., Anthelme F. (2008). Facilitation in plant communities: The past, the present, and the future. J. Ecol..

[34] Devagiri G.M., Khaple A.K., Mohan S., Venkateshamurthy P., Tomar S., Arunkumar A.N., Joshi G. (2016). Species diversity, regeneration and dominance as influenced by canopy gaps and their characteristics in tropical evergreen forests of Western Ghats, India. J. For. Res..

[35] Plotkin J.B., Potts M.D., Leslie N., Manokaran N., LaFrankie J., Ashton P.S. (2000). Species-area curves, spatial aggregation, and habitat specialization in tropical forests. J. Theor. Biol..

[36] Liu J., Feng C., Wang D., Wang L., Wilsey B.J., Zhong Z. (2015). Impacts of grazing by different large herbivores in grassland depend on plant species diversity. J. Appl. Ecol..

[37] Tilman D. (1982). Resource Competition and Community Structure.

[38] Sun C., Chai Z., Liu G., Xue S. (2017). Changes in species diversity patterns and spatial heterogeneity during the secondary succession of grassland vegetation on the Loess Plateau, China. Front. Plant Sci..

[39] Chen J., Huang D., Shiyomi M., Hori Y., Yamamura Y., Yiruhan (2007). Spatial heterogeneity and diversity of vegetation at the landscape level in Inner Mongolia, China, with special reference to water resources. Landsc. Urban Plan..

[40] Kéfi S., Rietkerk M., Alados C.L., Pueyo Y., Papanastasis V.P., ElAich A., De Ruiter P.C. (2007). Spatial vegetation patterns and imminent desertification in Mediterranean arid ecosystems. Nature.

[41] Li Y., Jiang Y., Shipley B., Li B., Luo W., Chen Y., Zhao K., He D., Rodríguez D., Chu C. (2021). The complexity of trait-environment performance landscapes in a local subtropical forest. New Phytol..

[42] Moeslund J.E., Arge L., Bøcher P.K., Dalgaard T., Ejrnæs R., Odgaard M.V., Svenning J.C. (2013). Topographically controlled soil moisture drives plant diversity patterns within grasslands. Biodivers. Conserv..

[43] Brockerhoff E.G., Ecroyd C.E., Leckie A.C., Kimberley M.O. (2003). Diversity and succession of adventive and indigenous vascular understorey plants in Pinus radiata plantation forests in New Zealand. For. Ecol. Manag..

[44] Zhao X., Feng Y., Xu K., Cao M., Hu S., Yang Q., Liu X., Ma Q., Hu T., Kelly M. (2023). Canopy structure: An intermediate factor regulating grassland diversity-function relationships under human disturbances. Fundam. Res..

[45] Johnson J.C., Williams C.J., Guertin D.P., Archer S.R., Heilman P., Pierson F.B., Wei H. (2021). Restoration of a shrub-encroached semi-arid grassland: Implications for structural, hydrologic, and sediment connectivity. Ecohydrology.

[46] Deng J., Chong Y., Dan Z., Kang D., Han X., Yang G. (2020). Aggregated Distribution of Herbaceous Plants in Restored Vegetation Community in a Semi-arid Area: Evidence from the Loess Plateau of China. Russ. J. Ecol..

[47] Han J., Wu N., Wu Y., Zhou S., Bi X. (2024). Spatial effects of shrub encroachment on wetland soil pH and salinity in the Yellow River Delta, China. J. Coast. Conserv..

